# Supplementary figures and images for: Approachability and Sensory Changes Following Mild Traumatic Brain Injury in Pigs
Source: Biomedicines. 2024 Oct 23;12(11):2427. doi: 10.3390/biomedicines12112427 (PMC11591678; doi:10.3390/biomedicines12112427)

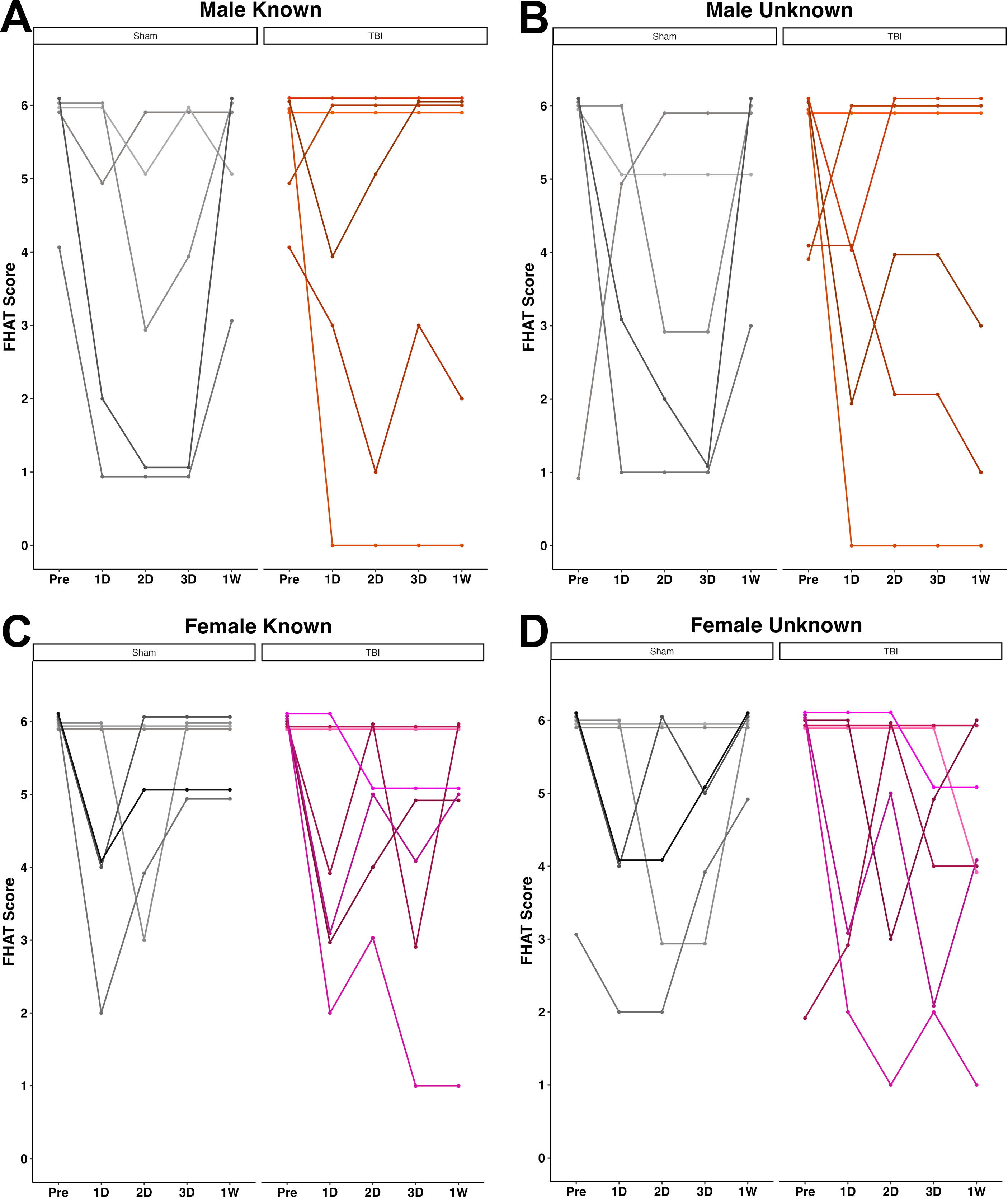

Supplement: Supplementary file 1 [file biomedicines-12-02427-s001.zip › Figure S1.tif]

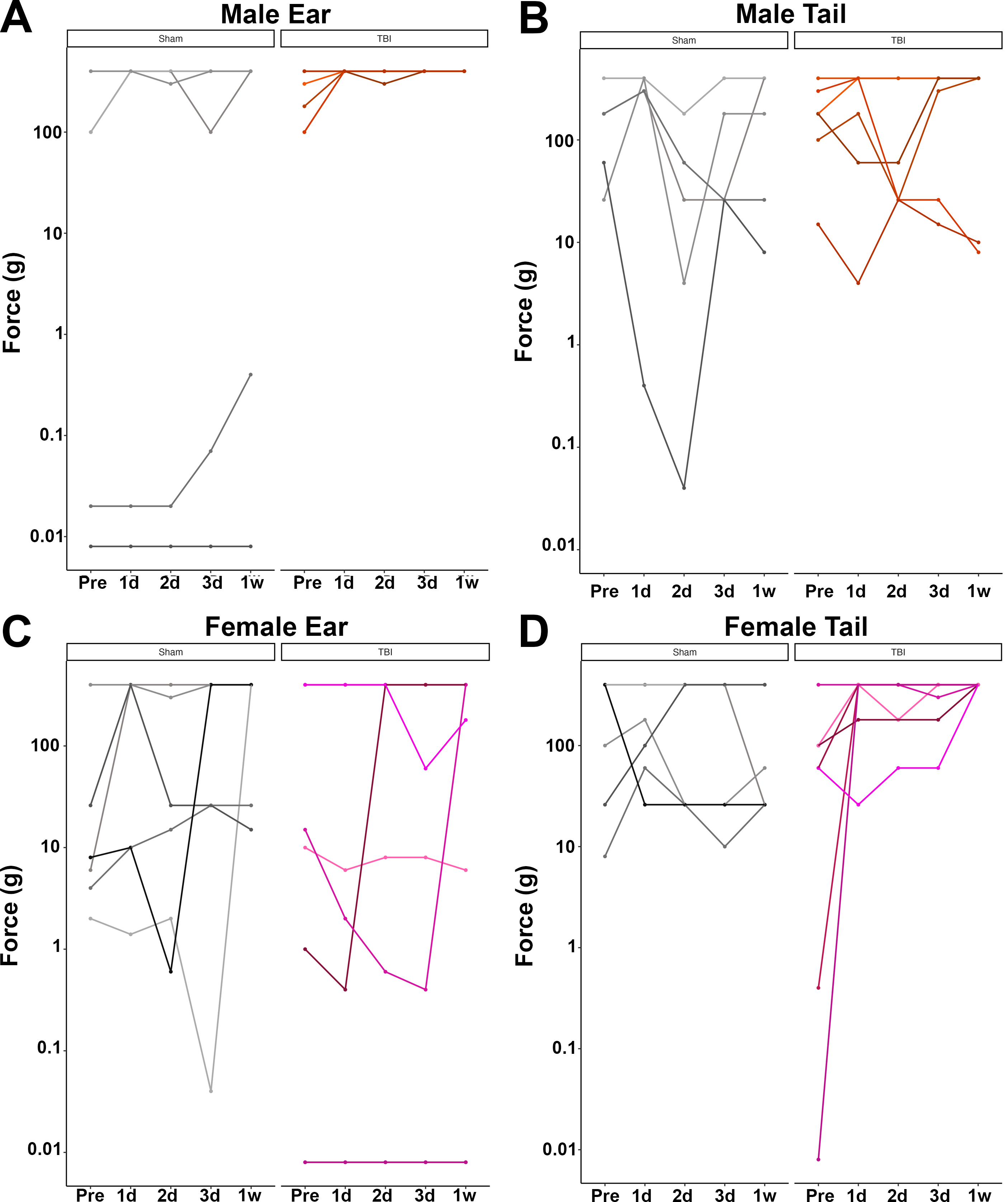

Supplement: Supplementary file 1 [file biomedicines-12-02427-s001.zip › Figure S2.tif]
